# Diffuse lamellar keratitis as a rare complication of diamond burr superficial keratectomy for recurrent corneal erosion: a case report

**DOI:** 10.1186/s12886-022-02589-3

**Published:** 2022-09-07

**Authors:** Hung-Yu Lin, Wei-Ting Ho

**Affiliations:** 1 Department of Ophthalmology, Far Eastern Memorial Hospital, No.21, Sec. 2, Nanya S. Rd., Banciao Dist., New Taipei City, 220 Taiwan; 2 School of Medicine, National Yang Ming Chiao Tung University, Hsinchu, Taiwan

**Keywords:** Laser in situ keratomileusis, Diffuse lamellar keratitis, Recurrent corneal erosion, Diamond burr superficial keratectomy, Case report

## Abstract

**Background:**

To present a case with a history of laser in situ keratomileusis (LASIK) developing diffuse lamellar keratitis (DLK) after diamond burr superficial keratectomy (DBSK) for recurrent corneal erosion (RCE).

**Case presentation:**

A 25-year-old man presented with multiple episodes of RCE one year after femtosecond-assisted LASIK for myopia correction. Because conservative treatments failed to halt the repetitive attack of RCE, he underwent epithelial debridement and DBSK. However, severe foreign body sensation and blurred vision developed on postoperative day one. The next day, slit lamp biomicroscopy revealed DLK manifested as diffuse granular infiltrates at the flap interface. After topical corticosteroid treatment, the inflammation resolved gradually, and his vision recovered to 20/20.

**Conclusions:**

Diffuse lamellar keratitis is a rare post-LASIK complication that can be triggered by DBSK, which causes impairment of the corneal epithelial integrity and subsequent inflammation at the flap interface. For post-LASIK patients with RCE, alternative treatments, such as anterior stromal puncture, may be considered to avoid extensive disruption of corneal epithelium and DLK development depending on the size and the location of the lesions.

## Background

Laser in situ keratomileusis (LASIK) is one of the most common corneal refractive surgeries to correct refractive error [1]. Despite well-established safety and efficacy, several postoperative complications may occur even after successful surgery [2, 3]. Diffuse lamellar keratitis (DLK) is an uncommon but potentially sight-threatening complication after LASIK and is elicited by epithelial injury during or after surgery [4]. In this report, we presented a case of recurrent corneal erosion (RCE) after femtosecond-assisted LASIK, who further developed acute DLK after using diamond burr superficial keratectomy (DBSK) to treat RCE.

## Case presentation

**Fig. 1:**
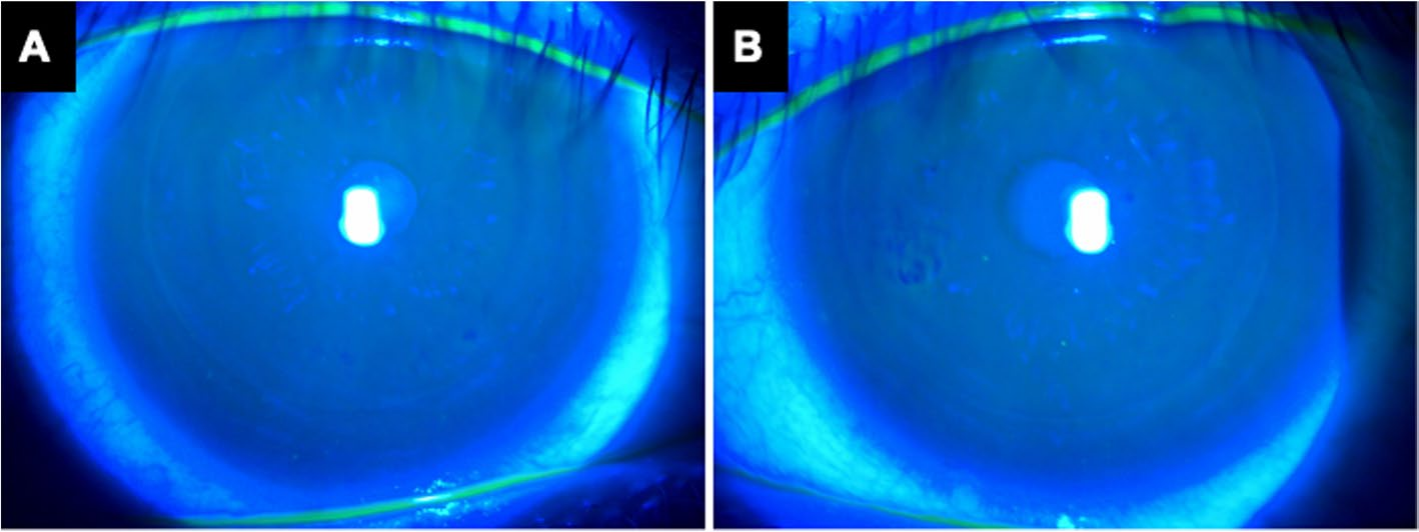
The external eye photographs before superficial diamond burr polishing. Fluorescein staining demonstrated intact epithelium with smooth surface in the right eye (**A**) and a patch of loosened epithelium at nasal paracentral area of the left eye (**B**)

A 25-year-old man who denied prior ocular surface diseases or trauma suffered from multiple episodes of RCE in the left eye one year after bilateral femtosecond-assisted LASIK to correct his myopia. Slit lamp biomicroscopy showed no epithelial basement membrane dystrophy in the right eye (Fig. 1A). Medical treatments, including topical antibiotics, preservative-free lubricant and tetracycline ointment were instilled but were unable to prevent further recurrences. In the last episode, the loosened epithelium was located in the paracentral cornea of the left eye that resulted in foreign body sensation and blurred vision (Fig. 1B). He then received epithelial debridement and DBSK within the LASIK flap margin. Extended-wear bandage soft contact lens and prophylactic topical antibiotics were applied postoperatively.

**Fig. 2:**
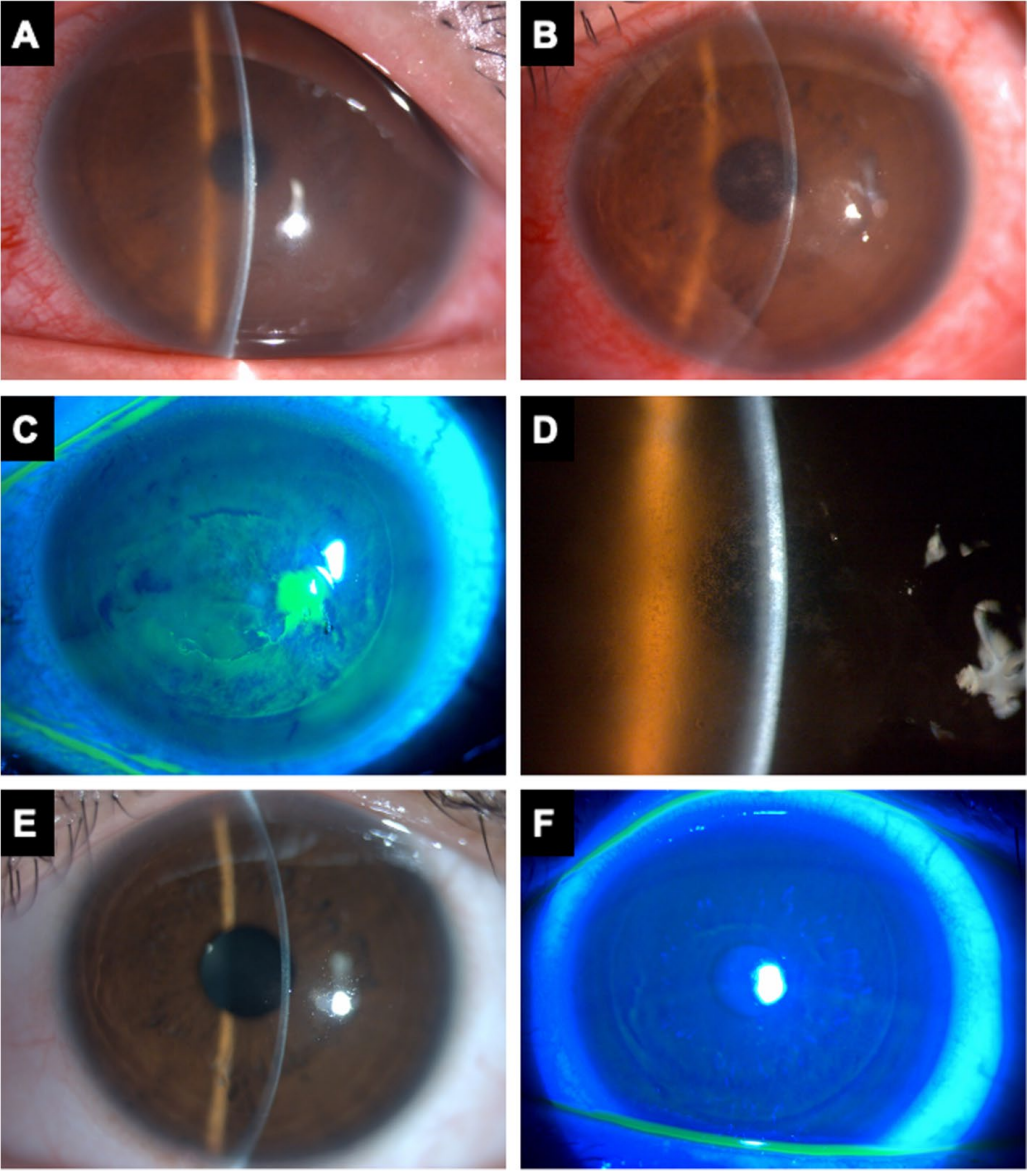
Diffuse lamellar keratitis after diamond burr superficial keratectomy. **A** The external eye photograph on postoperative day 1 after DBSK showed mild corneal edema and ciliary injection. **B-D** Increased grainy infiltrates at the flap interface involving the visual axis were noted on postoperative day 2. Stage 3 DLK is diagnosed and magnified view showed the “sands of the Sahara” appearance. **E–F** Reduced inflammation and disappearance of flap interface infiltration after topical corticosteroid treatment

On postoperative day 1, he presented to our clinic with foreign body sensation, redness and mild blurred vision in the left eye (Fig. 2A). The next day, his symptoms aggravated, and the best corrected visual acuity (BCVA) decreased to 20/60. Slit lamp biomicroscopy revealed conjunctival injection and severe corneal flap swelling. Diffuse grainy infiltrates were presented at the flap interface, which was reminiscent of “sands of Sahara” (Fig. 2B-D). Under the diagnosis of diffuse lamellar keratitis, he received preservative-free betamethasone and prophylactic antibiotic eye drops. Symptoms and signs gradually resolved 3 days after the treatment, and the BCVA recovered to 20/20 (Fig. 2E-F).

## Discussion and conclusion

Since its introduction in the late 1990s, LASIK has become one of the most popular refractive surgeries and enjoys the advantages of less postoperative discomfort, faster visual recovery and less corneal haze compared with previous photorefractive keratectomy [1]. One of the intraoperative complications of LASIK is epithelial defect or sloughing, which may predispose the patient to late onset RCE [3, 5]. With the advancement of surgical technique, LASIK flap creation by femtosecond laser has lower the incidence of epithelial sloughing due to lack of shearing force over the corneal epithelium as by microkeratome [6]. Although our case underwent femtosecond-assisted LASIK to correct myopia, he still developed late onset RCE without the evidence of epithelial basement membrane dystrophy in his fellow eye. This result demonstrated that RCE remains to be a potential postoperative complication after femtosecond-assisted LASIK.

Recurrent corneal erosion is characterized by weakened adhesion between epithelium basal cells and basement membrane at the level of hemi-desmosomes [7]. First-line treatments include topical lubricants, hypertonic saline, autologous serum eye drops, nighttime ointment and therapeutic bandage contact lens [7]. Nevertheless, the response rate is often suboptimal, ranging from 50 to 70% [8, 9]. If conservative management failed, surgical treatments, such as anterior stromal puncture, diamond burr superficial keratectomy (DBSK) or phototherapeutic keratectomy (PTK) should be considered [7, 10]. In our case, we chose DBSK for its simplicity and comparable successful rate to that of PTK [7, 9]. Furthermore, we only polished the loosened epithelium within the flap margin to avoid inadvertent flap dislocation. However, our case developed severe ciliary injection, flap swelling and diffuse grainy infiltration at the flap interface 2 days after surgery. The clinical appearance and rapid response to corticosteroid treatment confirmed the diagnosis of DLK. These evidences indicate that DBSK in post-LASIK patients may predispose to DLK.

Diffuse lamellar keratitis is one of the postoperative complications after LASIK [11]. Intense observation is extremely important in the first few days of DLK. In mild DLK, topical corticosteroid eye drops and ointment should be administered. Topical antibiotics or antiviral agents should be added initially if infectious etiology is suspected. In severe cases that centripetal migration of the inflammatory cells toward the central cornea leads to visual acuity deterioration and potential scar formation, flap lifting and irrigation of the interface are suggested to debulk the excessive inflammatory cells [12]. Due to the rapid response to preservative-free betamethasone eye drops in our case, further surgical intervention was avoided. While typical DLK occurs within one month postoperatively, late-onset DLK is relatively rare [13]. A case series of 12 eyes with DLK at least 6 months postoperatively demonstrated that viral infection, accidental or surgical trauma are the inciting factors [13]. Among the cases, one patient who received LASIK 15 years ago underwent superficial keratectomy for a 2 mm Salzmann’s nodule in right central cornea, and DLK subsequently developed [13]. In our case, we used DBSK to treat his RCE after femtosecond-assisted LASIK, which also resulted in late-onset DLK. Therefore, late-onset DLK should be identified as a potential complication after surgical manipulation of the ocular surface in post-LASIK patients. Intriguingly, both RCE and DBSK lead to disruption of epithelial integrity, but DLK was elicited by DBSK instead of RCE. In this patient, the RCE is microform according to Chandler’s classification [14]. The epithelial breakdown of the initial RCE is minimal compared with deliberate removal of loosened epithelium by DBSK that may allow the infiltration of exogenous materials into the flap interface. Even in the scenario of macroform RCE manifested as large epithelial defect, the LASIK flap-corneal stromal junction is less disturbed compared with DBSK. As a result, DBSK was a more direct trigger for DLK development in this patient.

As aforementioned, PTK and DBSK are among the surgical interventions to treat RCE. However, similar to DBSK, PTK will inevitably disrupt the corneal epithelium, causing keratocyte injury and chemotaxis of polymorphonuclear cells [15]. The leukocytic infiltration may further extend into the flap interface due to low tissue resistance, leading to DLK development [16]. On the contrary, anterior stromal micropuncture (ASP) by needle or Nd:YAG laser generates tiny adhesions between corneal epithelium and the stroma while avoiding extensive disruption of epithelium like PTK or DBSK [7]. In addition, the choice of treatment should also depend on the size and the location of the lesions [17]. For small lesions outside the visual axis, ASP may be considered to minimize recurrence while avoiding the rare complication of DLK. For large lesions or lesions in the visual axis, DBSK or PTK may still be warranted, but the potential complications, such as DLK in this case report, should be monitored.

In conclusion, DLK is a rare postoperative complication after LASIK, and can be triggered by DBSK for RCE in post-LASIK patient. Differentiation between DLK and infectious entity is crucial for successful management. Surgical treatments like DBSK or PTK for RCE may disrupt the epithelial integrity of LASIK flap junction in post-LASIK patient, thereby predisposing the patient to DLK. Alternative treatment strategies for RCE, such as ASP or conservative management, may be selected in this clinical scenario depending on the lesion size and location.

## Data Availability

All data generated or analyzed during this study are included in this published article.

## Abbreviations

LASIK: Laser in situ keratomileusis
DLK: Diffuse lamellar keratitis
DBSK: Diamond burr superficial keratectomy
RCE: Recurrent corneal erosion
BCVA: Best corrected visual acuity
PTK: Phototherapeutic keratectomy
ASP: Anterior stromal micropuncture
